# Psychophysiological Response Differences Between Advanced and Beginner Climbers and Fatigue Management

**DOI:** 10.3390/jfmk10010050

**Published:** 2025-01-28

**Authors:** Alejandro Padilla-Crespo, Vicente Javier Clemente-Suárez, Álvaro Bustamante-Sánchez

**Affiliations:** 1 Department of Sport Science, Faculty of Sport Sciences, Universidad Europea de Madrid, Calle Tajo, s/n, Villaviciosa de Odón, 28670 Madrid, Spain; alejandropadillacrespo@gmail.com (A.P.-C.); busta.es@gmail.com (Á.B.-S.); 2 Grupo de Investigación en Cultura, Educación y Sociedad, Universidad de la Costa, Barranquilla 080002, Colombia

**Keywords:** climbing, psychological factors, heart rate variability, grip strength, fatigue, anxiety, injury prevention

## Abstract

**Background/Objectives:** Rock climbing is a multifaceted athletic activity that requires both psychological and physiological resilience. This study aimed to examine the differences in psychological factors and fatigue predictors between novice and advanced climbers, with a focus on the interplay between experience and performance. **Methods:** The study included 60 participants categorized based on climbing experience (novice or advanced). Psychological and physiological assessments were conducted, including heart rate variability (HRV), grip strength, rate of force development (RFD), subjective perceived stress (SPS), and anxiety levels using validated questionnaires. **Results:** Advanced climbers exhibited lower anxiety levels and better sympathetic modulation compared to novices. Significant differences in HRV parameters, grip strength, and RFD were observed, reflecting the impact of experience on physiological responses. Advanced climbers demonstrated notable strength decreases post-climbing, supporting the utility of a force sensor on a 20 mm edge for assessing forearm fatigue. Correlations between cortisol levels, anxiety, and self-confidence during climbing were also identified. **Conclusions:** The findings highlight the importance of psychological and physiological factors in climbing performance. Forearm fatigue emerged as a critical predictor, suggesting that portable force sensors can optimize training and injury prevention. Insights from this study may enhance training protocols and improve real-time performance monitoring in climbers.

## 1. Introduction

Rock climbing is a multifaceted athletic pursuit characterized by intricate psychological and physiological challenges [1,2]. In the context of lead climbing, climbers must secure the safety rope by affixing it to anchors as they ascend the designated route. Failure to properly secure the safety rope may result in a relatively short fall. “The inability to generate and/or to sustain the finger force necessary to maintain contact with the hold is the main reason for an unsuccessful ascent or failure on a given climbing move” [3]. Studies suggest that lead climbing is not only a physically demanding activity but also a cognitive challenge that involves working memory [4], and it also tends to induce heightened perceived stress due to the augmented cognitive demands and the potential consequences associated with a fall [5]. Anxiety is a key factor in sports performance. It has been widely studied in sports psychology and can be differentiated into two states: state anxiety and trait anxiety. Moreover, self-concept and self-esteem have been identified as key determinants of satisfaction and performance in athletes and mountain technicians [6]. State anxiety refers to a situational and temporary response to a specific stimulus, such as a sports competition [7]. Trait anxiety, on the other hand, represents a stable personality disposition that predisposes individuals to perceive various situations as threatening and consequently experience higher levels of state anxiety [8]. Currently, this psychophysiological perspective can be analyzed through established indicators of emotional state supported by physiological stress parameters, personality characteristics, or questionnaires like the CSAI-2R or STAI, as observed in specific studies on climbing [5,8,9,10,11,12].

In this regard, heart rate variability (HRV) is a parameter that reflects the interactions between the heart, the brain, and the dynamics of the sympathetic nervous system (SNS) and the parasympathetic nervous system (PNS) [13]. Through HRV analysis, autonomic modulation can be assessed, serving as a reliable indicator of health and stress levels [14,15]. However, despite the study of heart rate (HR) and HRV being present in numerous sports, there is still much room for development in climbing. Previous research has assessed HR in lead climbing, but there are still no studies in this field that have examined the effects of this sport on autonomic modulation through HRV [11,12,16]. Similarly, we have not found previous works that assess fatigue at the level of the nervous system using another type of test like reaction time [11,12,16].

In sports performance research, psychological and physiological theories provide a crucial foundation for understanding how athletes manage stress, acquire skills, and optimize their performance. In this context, sport climbing can benefit from several widely recognized theoretical frameworks. The flow theory [17] highlights the importance of balancing challenge and skill, suggesting that flow states occur when task demands align with an individual’s abilities, promoting optimal engagement and execution. In contrast, the stress inoculation theory [18] posits that repeated exposure to stressors enables athletes to develop resilience, allowing them to better manage anxiety in challenging situations. Additionally, the cognitive appraisal theory [19] explains how the perception of a task influences emotional and physiological responses, as individuals continuously evaluate the relationship between task demands and their ability to cope. Finally, the deliberate practice theory [20] emphasizes that expert skill acquisition requires structured, effortful practice that involves progressively challenging tasks designed to address specific weaknesses.

Sport climbing is characterized by performing repeated isometric contractions of the finger flexors [21]. These contractions cause regular periods of ischemia (reduced blood flow) in the finger flexor muscles [2]. For that reason, forearm muscle strength has been acknowledged as a favorable performance indicator [2,21,22,23]. Fatigue in these muscles can lead to a decline in climbing performance, and strength, endurance, and cumulative fatigue of the finger flexor muscles may vary depending on the climber’s level [3,24,25]. It can significantly impact training planning, monitoring, and competition, emphasizing the need for further research.

Results from previous studies [3,25,26,27,28] confirm that forearm muscles experience fatigue during climbing, with a very significant decrease in grip strength observed from the beginning to the end of a climbing protocol. However, these studies have not analyzed local forearm fatigue using specific grip force sensors that involve the fingers in a specific climbing position [3,25,26,27,28].

Therefore, this study has two main objectives: to analyze the differences in psychological factors in sport climbing among novice and advanced climbers and to assess which type of test is a good predictor of fatigue in climbing. We hypothesized that advanced climbers will have a lower level of anxiety and sympathetic modulation than beginner climbers and that fatigue control in grip strength will be the best fatigue predictor.

## 2. Materials and Methods

### 2.1. Subjects

A sample of 60 climbers (33 with more than one year of experience and 27 with one month of experience) were included, consisting of 11 women and 49 men aged between 20 and 52 years with a 16.3 ± 3.16 IRCRA level (Table 1). The IRCRA level for beginner climbers was intermediate. The protocol was conducted in accordance with the ethical standards set forth in the Declaration of Helsinki under code CIPI/23.181, and it was approved by the Ethics Committee of Research at the European University. Inclusion criteria were being over 18 years old, knowing how to lead climb, and absence of an injury or condition that would advise against engaging in intense physical effort.

### 2.2. Procedure

The participants attended the corresponding climbing facility on a single day. They first completed the informed consent and provided demographic information (age, gender, height, weight, climbing performance, and experience). Subsequently, a standardized self-directed warm-up was conducted to increase the heart rate and muscle temperature and prepare the tissues for subsequent performance as part of the tests. First, they performed a mobility routine for the key joints involved in climbing (ankle, hip, shoulder, wrist, and fingers). Subsequently, they climbed on a vertical wall (15 m) using big holds. Afterward, they climbed a route two or three grades below their on-sight level and concluded the warm-up with a hangboarding exercise to properly activate finger flexors: 10 repeaters 7s hanging and 3s resting on a 20 mm rung.

**Figure 1:**
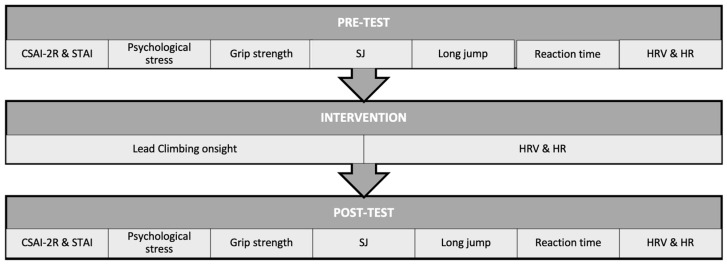
Organization of the intervention.

Subsequently, they performed initial tests (Figure 1) and then climbed a sport climbing route on-sight, choosing a difficulty level within their usual on-sight grade. Immediately after descending from the route, they repeated the initial tests. Considering that participants will never have exactly the same characteristics, we ensured that they were at least under the same conditions; they were asked to abstain from engaging in strenuous exercise 48 h before the tests as well as to avoid eating, consuming coffee or tobacco, or taking medications that could affect maximum physical effort 2 h before the tests.

### 2.3. Materials

Body weight was determined with participants barefoot and wearing shorts and an undershirt, and height was measured using the Frankfurt plane with an integrated stadiometer on the scale. A bioimpedance analyzer (InBody 720, Biospace Co. Ltd., Seoul, Republic of Korea) was used for body mass measurement (to the nearest 0.1 kg). A portable stadiometer (SECA, Leicester, UK) was used for the measurement of body height (to the nearest 1 cm). BMI was calculated as the quotient of body mass (kg) to height squared (m^2^).

The Competitive State Anxiety Inventory Second Revision (CSAI-2R) was used to assess anxiety and self-confidence. The CSAI-2R consists of 17 items that are scored on a Likert scale from 1 to 4, and the combined scores result in a final score for each of the 3 subscales (somatic anxiety, cognitive anxiety, and self-confidence). The European Spanish version of the CSAI-2R consists of 18 items [29].

The Spielberger Trait Inventory (STAI Form Y-2 test) was employed to evaluate state anxiety [10] with 20 statements referring to how a person generally feels; a higher total score reflected a higher anxiety trait (20–80 range).

Subjective perceived stress (SPS) was assessed using a 0–100 scale, with 0 representing the minimum they have ever felt in their life and 100 representing the maximum [30,31].

Grip strength: The maximum strength parameter used was maximum voluntary contraction (MVC), which was performed with the finger flexors on a climbing hold known as a “rung” measuring 20 mm in depth and attached to a force sensor (Chronojump force sensor, 24 bits, 160 Hz, Chronojump software 2.2.1. Barcelona, Spain). The same protocol as Giles et al. [32] was employed, involving three alternating attempts of a maximum five-second contraction with each hand (dominant and non-dominant), with climbers resting for one minute between attempts. The mean of the maximum value from each attempt in Newtons (N) was used for the statistical analyses. We also assessed RFD200ms, Time95%, and %MVC200ms [33,34,35,36].

SJ jump: This involved performing a jump from a semi-flexed knee position and subsequently making contact with the ground in the same position [37]. It has been demonstrated that vertical jump performance is affected by fatigue in various muscle groups [38,39]. Each athlete made 3 attempts, and the best result was recorded.

Long jump: The participants initiated the jump in a standing position, swinging their arms and flexing their knees to generate maximum forward propulsion. The jump length was determined using a measuring tape from the take-off line to the point of contact upon landing (i.e., the heels). Each athlete made 3 attempts, and the best result was recorded [40,41,42].

Critical flicker fusion threshold (CFFT): Participants sat in front of a display unit (Lafayette Instrument Flicker Fusion Control Unit, Model 12021., Lafayette, IN, USA). Within the display unit, two light-emitting diodes (58 cd/m^2^) were simultaneously presented, one for the left eye and another for the right eye. The stimuli were separated by 2.75 cm (center to center), with a stimulus–eye distance of 15 cm and a viewing angle of 1.9°. The interior of the display unit was painted black to minimize reflections. The flicker frequency increased (2 Hz/s) from 20 until the participant perceived fusion. After binocular fixation on the fovea, participants responded by pressing a button to identify the fusion thresholds (ascending frequency).

Before the experiment, participants underwent two practice trials to become familiar with the test requirements. Subsequently, three ascending trials were performed. The subjects took the test three times with a 5 s interval, and the best value was recorded [43,44,45].

Heart rate variability: HRV was recorded during the pre-tests, during the intervention, and during the post-tests. HRV was measured and analyzed in accordance with the recommendations of the Task Force of the European Society of Cardiology and the American Society of Pacing and Electrophysiology. The Polar H10 heart rate monitor (Polar, Kempele, Finland), known for its high-quality and reliable measurements, was used [46].

### 2.4. Statiscal Analysis

The SPSS statistical package (version 21.0; SPSS, Inc., Chicago, IL, USA) was used to analyze the data. Normality assumptions were checked with a Kolmogorov–Smirnov test. Descriptive statistics were presented as mean and standard deviation. A MANOVA (multivariate analysis of variance) test was used. The level of significance for all the comparisons was set at *p* < 0.05.

## 3. Results

The results are reported with their mean and standard deviation. In Table 1, the participants characteristics are shown. Table 2 shows the results of physical tests. None of the variables showed significant differences between groups or over time, except for Flicker, where differences were observed in the pre-test between beginners and advanced. Differences were also observed in the post-test, and additionally, there were differences between the pre-test and post-test in beginner climbers.

Table 3 and Table 4 display the results for grip strength and rate of force development (RFD) in both the dominant and non-dominant hands. Focusing on the dominant hand, significant differences were observed in the MVC parameter in the pre-test between beginner and advanced climbers. Similarly, in the post-test, significant differences were observed between beginners and advanced climbers. Additionally, there were significant differences between the pre-test and post-test in advanced climbers. In RFD200ms, there were differences in the post-test between beginners and advanced climbers. There were significant differences in the F200ms parameter in the pre-test between beginner and advanced climbers too. Similarly, in the post-test, significant differences were observed between beginners and advanced climbers. Additionally, there were significant differences between the pre-test and post-test in advanced climbers.

Regarding the non-dominant hand, significant differences were also observed in the MVC parameter in the pre-test between beginner and advanced climbers. Similarly, in the post-test, significant differences were observed between beginners and advanced climbers. Additionally, there were significant differences between the pre-test and post-test in advanced climbers. In RFD200ms, differences were observed in the pre-test between beginners and climbers. Differences were also observed between the pre-test and post-test in advanced climbers. In T’95%, differences were observed in the pre-test and post-test between beginners and advanced climbers. Finally, in F200ms, significant differences were observed in the pre-test between beginners and advanced climbers, in the post-test, and between the pre-test and post-test in advanced climbers. Additionally, there were differences between the pre-test and post-test in advanced climbers.

Table 5 shows the results of HR and HRV. There was a tendency toward significance in HR for beginner climbers for both pre-post, pre-int, and int-post. The same trend was observed in advanced climbers.

Regarding HRMax, significant differences were observed between groups of climbers in the post phase. There was also a tendency toward significance in beginner climbers, both in pre-post and int-post. Simultaneously, the same trend was observed in advanced climbers for int-post.

For the variable HRMin, significant differences were observed in beginner climbers for the pre-int and int-post phases and in advanced climbers at all moments.

Moving on to analyze heart rate variability parameters in the time domain (SDNN, RMSSD, and PNN50), we observed a trend towards significance in beginner climbers for pre-int and int-post and in advanced climbers for SDNN in pre-int. For the RMSSD parameter, there were significant differences between groups of climbers at the pre-moment and in beginners and advanced climbers in pre-int. For the PNN50 parameter, significant differences were observed between groups of climbers in post, in beginner climbers at all moments, and in advanced climbers in pre-post and int-post.

Regarding the parameters of heart rate variability in the frequency domain, significant differences were found in advanced climbers at all moments for the LF parameter except in pre-post. The HF parameter behaved similarly.

To conclude, regarding the parameters of heart rate variability in the non-linear domain, significant differences were observed between groups for the SD1 variable at the pre-moment and similarly in the pre-int moment for beginners and advanced climbers. For the SD2 variable, significant differences were observed between groups at the post-moment and in beginners and advanced climbers at all moments.

In Table 6, the results of psychological tests are presented, revealing significant differences in the post-test between beginners and advanced participants. Regarding somatic anxiety, significant differences were observed in post-test between beginners and advanced participants as well as between pre-test and post-test in advanced participants.

Cronbach’s alpha coefficients were obtained, showing reliability scores of 0.78 (for pre) and 0.81 (for post) for cognitive anxiety, 0.83 (for pre) and 0.77 (for post) for somatic anxiety, 0.82 (for pre) and 0.78 (for post) for self-confidence, and 0.81 (for pre) and 0.85 (for post) for state anxiety, all meeting acceptable standards.

## 4. Discussion

The objectives of this study were to analyze the differences in psychophysiological factors in climbing among novice and advanced climbers and to determine which test would be a good predictor of fatigue in climbing.

The first hypothesis was fulfilled, as our results showed differences in psychological and physiological response in novice climbers compared to advanced climbers (CFFT, HRV, subjective stress, and somatic anxiety). The second hypothesis of this study was also fulfilled, demonstrating that forearm local fatigue control appears to be the best predictor of fatigue in climbing.

We did not find significant differences in the lower extremity strength tests. These results may be because the muscular groups required in on-sight sport climbing are not crucial in affecting fatigue. This reinforces the findings of various authors who claimed that the deep finger flexor muscle is the most important for climbing performance [21,47,48,49]. However, it would be interesting to apply these same tests to climbers who practice speed climbing, as lower body musculature plays a more determinant role in performance in this discipline [50,51].

An elevation in CFFT indicates heightened cortical arousal and improved information processing. Conversely, when values drop below the baseline, it indicates decreased efficiency in information processing and fatigue of the central nervous system [52]. We observed this latter behavior in the novice climbers in our study, suggesting fatigue in their cortical arousal after a stressful and demanding cognitive effort. This is the first study in climbing that has analyzed this parameter. However, in other sports in which experience is crucial to control cognitive stress, such as diving, a similar behavior has been found [53]. Although the practice of any sport may induce physiological fatigue by itself, the difference in CFFT response between novice and advanced climbers seems to be crucial because of the potential risks that this sport has in the perception of novice climbers. Future research should consider these findings to better assess the perception of novice climbers when they first practice this sport.

The upward trend in HR for pre-, int-, and post-climbing was observed in both advanced and novice climbers, and this trend is like that found in other studies [11,16,54]. The elevation of heart rate during climbing is attributed to various factors, some of which are challenging to quantify accurately. Anxiety stemming from acrophobia, as noted by Billat et al. [55], can lead to heightened muscle tension during climbing. This tension, in turn, can elevate blood pressure, consequently increasing heart rate, as observed by Goddard and Neumann [56]. Factors such as fear of heights, isometric force exertion, shifts in arm positioning, and heightened abdominal muscle tension can contribute to the escalation of heart rate during climbing. Consequently, relying on heart rate as a reliable indicator of climbing intensity is questionable [54]. The differences found in HR peak between novice and advanced climbers coincide with the study of Janot et al. [57], which discovered that novice climbers exhibited elevated heart rates compared to more experienced climbers. Fryer et al. [11] found a similar HR behavior when comparing lead climbing versus top climbing, with HR being higher in lead climbing. This could be due to the increased time spent in isometric contractions [58,59,60]. Regarding the parameters of heart rate variability in the time domain, a downward trend was observed in the parameters of SDNN, RMSSD, and pNN50 for both groups of climbers in pre-int and an upward trend in int-post. These results indicate that climbers present a lower level of stress before and after climbing, while there is increased sympathetic activity during climbing. In racket sports, a similar behavior has been observed, with a downward trend in these parameters regarding especially stressful sets [61,62,63]. This trend is also similar in military contexts, implying working in altitude, in which any error can result in fatal consequences [40,64]. The significant differences between beginner climbers and advanced climbers for the RMSSD parameter before climbing and pNN50 after climbing may be due to advanced climbers being more accustomed to these situations and having lower stress levels before climbing compared to a beginner climber. Advanced climbers exhibit significantly lower RMSSD values pre-climb and lower pNN50 values post-climb compared to beginner climbers. This difference indicates that experience plays a vital role in autonomic modulation, allowing advanced climbers to better manage the stress associated with climbing activities. Even so, it is worth noting that, on some occasions, emotional intelligence and resilience have been shown to be significant predictors of effective stress management in sports contexts, and this could influence the results [65].

The parameters of heart rate variability in the frequency domain, such as LF associated with modulation of the parasympathetic response, decrease while climbing, indicating increased sympathetic activity, as other authors have also shown in their research, where an increase in stress on the system led to a decrease in high-frequency band power [66].

Regarding the parameters of heart rate variability in the non-linear domain, a reduction in the SD1 and SD2 parameters was observed while climbing with a slight increase after climbing but without reaching the initial values in advanced climbers and beginners. This indicates good sympathetic adaptation to climbing, as this parameter is indicative of the long-term variability and modulation of the parasympathetic nervous system [14] and allows us to verify how athletes are able to respond appropriately to the stressful situation posed by challenges [66]. Our study data on SD1 and SD2 shows the influence of being aware of the “challenge” faced by expert climbers, without the help of instructors and with greater difficulty in the routes, as this can affect their prior autonomic modulation. Unlike what occurs in SD1 and SD2 in beginner climbers, if we observe the behavior of RMSSD again, it can be seen that although advanced climbers started lower than beginner climbers, the route affected them less afterwards (there were no differences between the pre- and during-moments in advanced climbers, but there were in beginner climbers). This is possibly due to the fact that advanced climbers are more aware of the challenge of their more difficult route without any assistance from an instructor during the pre-moment, so the route does not affect them as much as it does beginner climbers when comparing pre- and during-moments.

The significant differences in perceived subjective stress observed after climbing indicate an increase in this variable for beginner climbers. It shows that for beginner climbers, a sport climbing route produced a state of activation equal to or greater than the instant before climbing. However, the opposite occurred with advanced climbers. For the CSAI-2R questionnaire, the score range for each factor was calculated by summing the scores of the corresponding items, with a Likert scale from 1 to 4. The score ranges for each factor were as follows: cognitive anxiety: this factor included five items, each scored on a scale from 1 to 4, with a score range from 5 (minimum) to 20 (maximum); somatic anxiety: this factor included seven items, each scored on a scale from 1 to 4, with a score range from 7 (minimum) to 28 (maximum); and self-confidence: this factor included 5 items, each scored on a scale from 1 to 4, with a score range from 5 (minimum) to 20 (maximum). These ranges allowed assessment of the intensity of each factor in participants, providing a clear view of their levels of cognitive anxiety, somatic anxiety, and self-confidence. Regarding the interpretation of score levels, there is no universally established categorization to define low, moderate, and high levels; therefore, according to ref. [29], the most accurate approach would be to compare each result with other articles. Regarding somatic anxiety, significant differences were observed after climbing in advanced climbers, showing higher anxiety before climbing. This did not occur in beginner climbers, where this anxiety was similar before and after climbing, as also observed in the study by Villavicencio et al. [11] with a sample of intermediate climbers, which could be justified by the additional stress caused by sport climbing for non-advanced climbers. Comparing the response of anxiety to different types of climbing, Hodgson et al. [5] found significant differences between top rope and lead climbing for somatic anxiety in intermediate climbers who experience higher levels of anxiety during lead climbing compared to top rope climbing. Previous research has reported that anxiety negatively affects climbing efficiency. This includes increased contact time with holds [67], a higher frequency of exploratory movements, extended climbing duration [68], and the need to use more holds to advance [69,70], especially in on-sight climbing. However, when evaluating changes in geometric entropy with repeated ascents on the same route, it has been observed that geometric entropy decreases, indicating lower variability and greater efficiency in movements. Climbers optimize their motor patterns as they become familiar with the route, suggesting a process of learning and adaptation. This improvement in efficiency is also associated with reduced energy demand during subsequent ascents [71]. But in lead climbing, the fear of falling is a critical factor that can limit climbing performance, especially in women [72]. This climbing performance might be improved through a reduction in anxiety and an increase in self-confidence [73]. Some studies show that a psychological training intervention appears to reduce anxiety levels and improve climbing ability, self-confidence, and interoceptive awareness in women climbers who have a fear of falling [72].

The low anxiety levels observed in both groups may be interpreted through the flow theory [17], suggesting that the climbing task might not have been optimally challenging to fully engage participants. This highlights the importance of task difficulty alignment in future studies. The stress inoculation theory [18] offers an explanation for the lower anxiety levels in experienced climbers, likely resulting from repeated exposure to climbing stressors and the development of resilience. The cognitive appraisal theory [19] provides a lens to understand how experienced climbers perceive the climbing challenge as less threatening, leading to reduced cognitive and somatic anxiety. Finally, the deliberate practice theory [20] underscores the need for progressively challenging training to induce the anxiety levels necessary for skill differentiation. In addition, the application of the flow theory, stress inoculation theory, cognitive appraisal theory, and deliberate practice theory offers a comprehensive framework for understanding climbers’ psychological responses. Another aspect to consider is that this intervention was carried out in a recreational/leisure context, and recreational sports activities are more associated with positive emotions and low levels of anxiety due to the absence of performance expectations and a greater perception of enjoyment and freedom compared to competitive situations. In contrast, competitive contexts generate state anxiety due to external evaluation and pressure for results [74]. Future studies should design tasks that better align with climbers’ skill levels and employ training protocols grounded in deliberate practice to optimize performance and stress management.

However, even though the difficulty of the climb is marked by a numerical grade, which is usually defined by the height of the route, the wall’s inclination, the size of the holds, and the difficulty of the movements, among other factors, it remains a “subjective” difficulty within the most objective parameters possible. The same route may pose a different challenge and provoke different anxiety and stress responses in two climbers due to differences in technical level, strength levels, height, psychological factors, and other aspects. Even working memory appears to be especially relevant in more complex situations, such as technical routes or boulder problems that require specific strategies [4]. In this study, it is important to remember that the climbers were ascending a route at their usual maximum on-sight grade. Even though the climbing test was chosen to ensure that all climbers were in the same situation and conditions, anxiety and stress responses may be influenced by two theories. Flow states, according to Mihaly Csikszentmihalyi’s flow theory [17], occur when the difficulty of a task aligns well with a person’s skill level, leading to optimal performance and engagement. If the test difficulty was not challenging enough for some climbers, they might not have experienced a high enough level of anxiety to influence their performance or differentiation. Similarly, Anders Ericsson’s deliberate practice theory applies to climbing [20]. In this sport, deliberate practice involves progressively challenging climbs that push cognitive and physical limits. If the test difficulty did not meet the necessary level for deliberate practice, the climber may not have experienced sufficient anxiety to highlight skill differences.

The observed differences in MVC in both hands between novice and advanced climbers align with values from similar studies comparing strength between recreational climbers versus non-climbers or elite climbers versus novice climbers versus non-climbers, with consistently higher strength values shown for the sample of climbers or advanced climbers, as they have already experienced the supercompensation effect on their finger flexor muscles after a concrete stressor like lead climbing [75,76]. In our study, the strength values significantly decreased after climbing in the advanced climber group, thus demonstrating that a pull with a force sensor on a 20 mm edge can be a valid test to monitor local forearm fatigue in advanced climbers. Fieldmann et al. [25] also observed a strength loss in MVC of 18% after climbing in a sample of elite climbers. The difference is that they measured it on a 23 mm edge with only one hand and after a high-intensity climbing test (HIT). In our study, we observed a strength loss of 10% for the dominant hand and 7% for the non-dominant hand. The difference in strength loss between our study and the results of Fieldmann et al. [25] may be due to the type of climbing test, with high-intensity climbing being a more demanding exercise that accumulates more waste substances, such as lactate and inorganic phosphate, and consequently increases fatigue and the loss of strength after climbing. Watts et al. [3] analyzed the metabolic effects of a difficult climb in a group with active rest and another group with passive rest. In the active rest group, a significant decrease in grip strength was observed. However, grip strength was measured using a hand dynamometer instead of utilizing a fingerboard with a force sensor to simulate the specific climbing gesture. In our study, no decrease in grip strength was observed after climbing in the novice climber group. This could be because the beginner group climbed routes of lower difficulty. Such routes are characterized by having handholds with greater depth, which entail less proportional recruitment from the flexor digitorum profundus muscle (FDP) [77,78], which is considered the most important muscle in climbing performance [21,47,48,49]. Another variable to consider is the edge size used for the force sensor test, as this can affect the reliability of the measurement. It appears that very small or large edges result in greater variability in the outcomes, which limits their accuracy in evaluating grip strength. For this reason, considering the climbing level of the participants, we decided to conduct the test on a medium-sized edge, as suspension tests on intermediate edges seem to be the most suitable for distinguishing between different performance levels [79].

Research has consistently shown that rate of force development (RFD) and maximal force are key factors in sport climbing performance [33,34,35,36]. Concerning the RFD200ms, this metric may be employed with high precision when comparing individuals either within or across subjects for training purposes [34]. The differences observed in RFD200ms, T’95%, and F200ms between beginner and advanced climbers align with the values reported in other studies. Specifically, Stien et al. [35] found no differences between intermediate and advanced climbers, but the elite group exhibited significantly higher RFD and force output compared to both other groups. Levernier et al. [36] also noted differences in RFD200ms for all conditions: 24.51% between international climbers and skilled climbers, 34.04% between non-climbers and skilled climbers, and 50.21% between international climbers and non-climbers. Regarding F200ms, a significant decrease was observed in the group of advanced climbers for both the dominant and non-dominant hands, with the same trend observed for the RFD200ms parameter but only in the non-dominant hand. The significant reduction in two RFD parameters for the non-dominant hand coincides with the findings of Giles et al. [32], who determined that regardless of skill level and gender, the reoxygenation velocity of FPD is lower in the non-dominant hand, specifically 13.6% lower compared to the dominant hand. Once again, this information suggests that performing a pull with the non-dominant hand could be a valid test to assess forearm fatigue at a local level for advanced climbers. However, further studies are needed to confirm this finding, and it would be interesting to have a larger sample size, as this could be a limitation of this study.

## 5. Conclusions

This study highlights the importance of psychological factors in climbers, particularly beginners, as shown by decreased CFFT levels, indicating lower information processing efficiency and central nervous system fatigue. Beginner climbers also experience higher perceived stress and somatic anxiety compared to advanced climbers, with a climbing route inducing stress levels similar to or greater than pre-climb moments.

HRV data show that sport climbing significantly increases sympathetic activity and stress during the route compared to before and after in both groups. These parameters offer a valuable tool for coaches and researchers to monitor and control climbers’ psychophysiological responses more precisely.

For the second objective, performing an MVC test on a 20 mm edge with the non-dominant hand using a force sensor and evaluating strength loss or rate of force development (RFD) post-climb appears valid for forearm fatigue assessment, particularly for medium to small holds engaging the flexor digitorum profundus. Lower-body-focused physical tests are unreliable for fatigue detection in climbers. However, further studies are needed to corroborate this in the speed climbing modality.

This finding is crucial for preventing overuse injuries and optimizing training stimuli by adjusting training volume, as individual variability in maximum training volume at relative intensity is significant. Portable force sensors, now affordable and compatible with smartphones, can be easily used indoors or outdoors by climbers and coaches.

## Figures and Tables

**Table 1:** Characteristics of participants.

	Experienced Climbers (n = 33)	Beginner Climbers (n = 27)
Age (years)	33 ± 8.24	22 ± 1.65
Height (m)	1.73 ± 6.45	1.74 ± 9.72
Weight (kg)	66.4 ± 7.55	69.85 ± 10.23
IMC (kg/m^2^) ^1^	21.9 ± 1.76	22.98 ± 2.04
Climbing performance ^2^	16.3 ± 3.16	10
Lead experience (years)	6.3 ± 5.97	
Climbing modality (%lead)	100%	100%

The values are presented as mean ± standard deviation. ^1^ IMC = body mass index (kg/m^2^), ^2^ The highest grade achieved in climbing according to the classification system proposed by the International Rock Climbing Research Association (IRCRA).

**Table 2:** Results of physical tests.

	Experienced Climbers				Beginner Climbers						
	Pre		Post		Pre		Post				
	M	SD	M	SD	M	SD	M	SD	F	*p*	η^2^
MVC	487.18 *^,‡^	95.24	453.19 *	100.66	291.38	71.09	284.47	75.23	7.984	0.006	0.121
MVC/BW	0.745 *^,‡^	0.126	0.695 *	0.14	0.424	0.758	0.413	0.079	7.834	0.007	0.119
MVC/BW/mm	0.037 *^,‡^	0.006	0.034 *	0.006	0.021	0.003	0.020	0.003	8.009	0.006	0.121
RFD200ms	796.91 *^,‡^	196.79	734.23 *	208.44	392.97	187.75	354.58	211.79	0.403	0.528	0.007
T’95%	0.501 *	0.185	0.555 *	0.252	0.349	0.172	0.318	0.164	2.717	0.105	0.045
F200ms	351.84 *^,‡^	94.20	311.24 *	98.58	237.51	59.77	238.58	63.21	7.175	0.010	0.110

Note: M = Median; SD = standard deviation; F = Fisher–Snedecor test; η^2^ = partial eta-squared; RT = reaction time. * Differences between groups (*p* < 0.05). ‡ Differences between pre-post samples (*p* < 0.05).

**Table 3:** Results of finger strength test in the dominant hand.

	Experienced Climbers				Beginner Climbers						
	Pre		Post		Pre		Post				
	M	SD	M	SD	M	SD	M	SD	F	*p*	η^2^
SJ (cm)	28.79	5.76	29.16	7.20	29.98	6.98	31	7.23	0.458	0.489	0.008
Long Jump (m)	1.89	0.28	2.5	3.8	1.96	0.27	1.97	0.27	0.752	0.389	0.013
RT (s)	331.22	74.44	321.17	36.94	323.27	31.73	320.47	33.89	0.173	0.679	0.003
Flicker (s)	37.19 *	3.15	36.57 *	4.04	41.20 ^‡^	4.64	39.78	4.76	1.942	0.169	0.032

MVC: maximum voluntary contraction expressed in newtons; RFD200ms: RFD achieved at 200 ms expressed in newtons per second; T’95%: time to reach 95% of MVC expressed in seconds; F200ms: strength reached at 200ms expressed in newtons. Note: M = median; SD = standard deviation; F = Fisher–Snedecor test; η^2^ = partial eta-squared. * Differences between groups (*p* < 0.05). ‡ Differences between pre-post samples (*p* < 0.05).

**Table 4:** Results of finger strength test in the non-dominant hand.

	Experienced Climbers				Beginner Climbers						
	Pre		Post		Pre		Post				
	M	SD	M	SD	M	SD	M	SD	F	*p*	η^2^
MVC	513.80 *^,‡^	103.57	463.24 *	97.42	320.31	80	311.79	81.61	11.762	0.001	0.169
MVC/BW	0.782 *^,‡^	0.13	0.709 *	0.13	0.467	0.09	0.453	0.09	10.025	0.002	0.147
MVC/BW/mm	0.039 *^,‡^	0.006	0.035 *	0.006	0.023	0.004	0.022	0.004	11.882	0.001	0.170
RFD200ms	975.26	10,001.2	697.76 *	250.54	502.88	173.45	496.09	175.45	1.823	0.182	0.030
T’95%	0.507	0.323	0.554	0.411	0.43	0.175	0.529	0.287	0.241	0.626	0.004
F200ms	378.74 *^,‡^	105.37	330.96 *	115.03	246.20	62.32	220.66	75.86	1.469	0.230	0.025

Note: M = MVC: maximum voluntary contraction expressed in newtons; RFD200ms: RFD achieved at 200 ms expressed in newtons per second; T’95%: time to reach 95% of MVC expressed in seconds; F200ms: strength reached at 200 ms expressed in newtons. M = median; SD = standard deviation; F = Fisher–Snedecor test; η^2^ = partial eta-squared. * Differences between groups (*p* < 0.05). ‡ Differences between pre-post samples (*p* < 0.05).

**Table 5:** Results of autonomic modulation.

	Experienced Climbers						Beginner Climbers								
	Pre		Int		Post		Pre		Int		Post				
	M	SD	M	SD	M	SD	M	SD	M	SD	M	SD	F	*p*	η^2^
Average HR	89.78	11.94	140.15	20.64	97.15	15.25	91.44 ^‡‡^	13.69	146.77 ^‡^	20.41	95.92 ^‡‡^	13.38	2.446	0.091	0.040
Maximum HR	154.93	26.48	171.81 ^‡‡‡^	15.43	142.09 *	16.90	172.96 ^‡‡^	57.54	183.48 ^‡‡‡^	65.01	155.29	27.69	0.137	0.872	0.002
Minimum HR	61.57 ^‡‡^	7.58	93.42 ^‡‡‡^	23.80	70.81 ^‡^	14.22	63.96 ^‡‡^	9.58	91.07 ^‡‡‡^	19.08	67.88	10.78	0.882	0.417	0.015
SDNN	55.64 ^‡‡^	15.71	27.70	22.07	44.46	29	67.74 ^‡‡^	30.32	31.86 ^‡‡‡^	34.73	77.90	120.37	1.338	0.266	0.023
RMSSD	49.32 *^,‡‡^	25	32.20	29.11	37.91	30.97	67.76 ^‡‡^	41.42	38.05	38.27	54	31.66	1.198	0.306	0.020
PNN50	13.45 ^‡‡^	11.41	3.79	5.70	7.2 *^,‡^	9.76	20.27 ^‡‡^	15.67	6.81 ^‡‡‡^	13.06	14.88 ^‡^	13.43	1.490	0.230	0.025
LF (n.u.)	70.58 ^‡‡^	15.69	60.87 ^‡‡‡^	20.46	75.23	18.91	64.10	14.85	60.98	20.94	67.19	14.11	1.300	0.277	0.022
HF (n.u.)	29.36 ^‡‡^	15.67	39.01 ^‡‡‡^	20.39	24.72	18.89	35.81	14.81	38.91	20.85	32.74	14.08	1.299	0.277	0.022
SD1	34.89 *^,‡‡^	17.69	22.79	20.59	26.82	21.91	47.98 ^‡‡^	29.38	26.95	27.11	38.21	22.39	1.197	0.306	0.020
SD2	69.77 ^‡‡^	17.12	30.96 ^‡‡‡^	24.51	52.10 *^,‡‡‡^	26.39	82.10 ^‡‡^	33.46	34.89 ^‡‡‡^	41.75	67.95 ^‡^	28.98	1.180	0.311	0.020

Note: Int: intervention. M = median; SD = standard deviation; F = Fisher–Snedecor test; η^2^ = partial eta-squared; RMSSD = square root of the mean of the sum of the squared differences between adjacent normal R-R intervals; pNN50 = the percentage of differences between R-R intervals higher than 50 ms; HF = high frequency; LF = low frequency; n.u. = normalized unit; SD1 = Poincaré plot index of instantaneous recording of the variability; SD2 = Poincaré plot index of overall variability. * Differences between groups (*p* < 0.05). ‡ Differences between pre-post samples (*p* < 0.05). ‡‡ Differences between pre-intervention samples (*p* < 0.05). ‡‡‡ Differences between intervention-post samples (*p* < 0.05).

**Table 6:** Results of psychological tests.

	Experienced Climbers				Beginner Climbers						
	Pre		Post		Pre		Post				
	M	SD	M	SD	M	SD	M	SD	F	*p*	η^2^
Stress	35	24.89	29.33 *	23.14	43.74	18.19	49.29	23.92	4.931	0.030	0.078
Cognitive anxiety	9.09	2.77	8.42	3.03	10.22	2.87	9.37	3.54	0.045	0.834	0.001
Somatic anxiety	13.51 ^‡^	3.96	11.57 *	3.68	14.74	4.07	14.62	4.45	1.852	0.179	0.031
Self-confidence	15.21	2.66	15.54	3.12	15.66	3.37	16.40	3.33	0.158	0.692	0.003
STAI	17.75	5.20	15.69	8.91	18.51	6.41	18.62	10.11	1.084	0.302	0.018

Note: M = median; SD = standard deviation; F = Fisher–Snedecor test; η^2^ = partial eta-squared. * Differences between groups (*p* < 0.05). ‡ Differences between pre-post samples (*p* < 0.05).

## Data Availability

Data are available upon reasonable request.
